# Antidepressant-Like Effects of the Ethyl Acetate Soluble Fraction of the Root Bark of *Morus alba* on the Immobility Behavior of Rats in the Forced Swim Test

**DOI:** 10.3390/molecules19067981

**Published:** 2014-06-12

**Authors:** Dong Wook Lim, Yun Tai Kim, Ji-Hae Park, Nam-In Baek, Daeseok Han

**Affiliations:** 1 Food Resource Research Center, Korea Food Research Institute, Seongnam 463-746, Korea; E-Mail: neodw4015@kfri.re.kr; 2 Research Group of Food Functionality, Korea Food Research Institute, Seongnam 463-746, Korea; E-Mail: ytkim@kfri.re.kr; 3 Graduate School of Biotechnology and Research Institute of Life Sciences & Resources, Kyung Hee University, Yongin 446-701, Korea; E-Mails: wlgo3411@hanmail.net (J.-H.P.); nibaek@khu.ac.kr (N.-I.B.)

**Keywords:** *Morus alba*, antistress, antidepressant activity, forced swimming test

## Abstract

In this study, the antidepressant-like effects of *Morus alba* fractions in rats were investigated in the forced swim test (FST). Male Wistar rats (9-week-old) were administered orally the *M. alba* ethyl acetate (EtOAc 30 and 100 mg/kg) and *M. alba* *n*-butanol fractions (*n*-BuOH 30 and 100 mg/kg) every day for 7 consecutive days. On day 7, 1 h after the final administration of the fractions, the rats were exposed to the FST. *M. alba* EtOAc fraction at the dose of 100 mg/kg induced a decrease in immobility behavior (*p* < 0.01) with a concomitant increase in both climbing (*p* < 0.05) and swimming (*p* < 0.05) behaviors when compared with the control group, and *M. alba* EtOAc fraction at the dose of 100 mg/kg decreased the hypothalamic-pituitary-adrenal (HPA) axis response to the stress, as indicated by an attenuated corticosterone response and decreased c-fos immunoreactivity in the hippocampal and hypothalamic paraventricular nucleus (PVN) region. These findings demonstrated that *M. alba* EtOAc fraction have beneficial effects on depressive behaviors and restore both altered c-fos expression and HPA activity.

## 1. Introduction

Mental disorders are prevalent among the general population [1]. At any given time, approximately 10% of adults are experiencing a current mental disorder, and 25% will develop one at some point during their lifetime [2]. Depression is one of the most common mental disorders, affecting nearly 21% of the World’s population [3]. Despite recent progress in the development of clinically relevant anti-depressant drugs, the currently available anti-depressants are not totally effective, and are associated with many undesirable adverse effects [4]. Therefore, alternative approaches, including dietary interventions, have been of interest in the control of depression [5].

Continued and elevated glucocorticoid levels resulting from dysfunction of the hypothalamic-pituitary-adrenal (HPA) axis is one of the most prominent neurobiological findings in depression [6]. Glucocorticoid receptors (GR) mediate the direct effects of the glucocorticoids that are released in response to stress and regulate the HPA axis via a negative feedback mechanism [7]. Previous clinical studies have reported that depressed subjects exhibit down-regulation of GR expression, which subsequently leads to an increase in the endogenous levels of glucocorticoids [8]. Thus, GR function may be one of the potential mechanisms underlying HPA axis dysfunction [9].

Consistent with a role for glucocorticoids in depression, GR antagonists have been encouraged as having potential therapeutic benefits for stress-related disorders. This is based on the ability of GR antagonist to block the increase in the levels of circulating glucocorticoids and on their ability to up-regulate GR [10]. Recently, we have investigated the anti-glucocorticoid activity of the traditional Korean medicine using hormone-responsive element reporter assay [11], and the root bark of *Morus alba* extracts significantly decreased luciferase activity in response to cortisol in a concentration-dependent manner (unpublished data). Our finding demonstrated that the root bark of *Morus alba* extracts possesses potent antagonistic activity against glucocorticoid, at least in part.

Mulberry tree (*Morus alba* L.), one of the best known and most widely distributed trees or shrubs of the family Moraceae, whichis extensively cultivated in East Asia. The root bark of *M. alba* has been used in Oriental medicine for anti-inflammatory, diuretic and antipyretic purposes, and as a sedative [12]. *M. alba* extracts has also been reported to possess hypotensive [13], hypoglycemic [14], anti-inflammatory [15], and adaptogenic [16] effects.

In the present study, the antidepressant-like effects of *M. alba* fractions were investigated in rats in the forced swim test (FST), which may be used as an antidepressant activity model [17]. Moreover, to determine the neurobiological effects underlying the antidepressant-like activity of the *M. alba* fractions, corticosterone responses and c-fos immunoreactivity were evaluated in rats exposed to FST.

## 2. Results

### 2.1. Effects of M. alba Ethyl Acetate Soluble Fraction on Depressant Behaviors in Response to the FST

**Figure 1:**
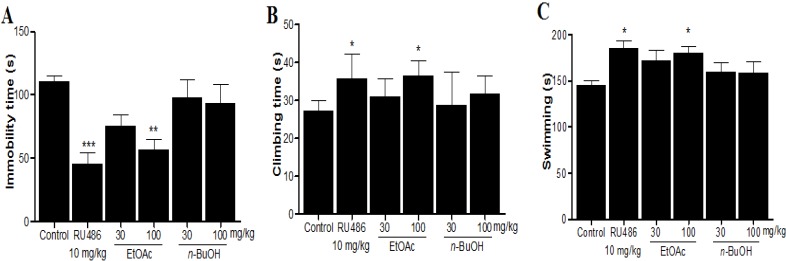
Antidepressant-like effects of treatments with *M. alba* fractions on depressive behavior in response to the FST. *M. alba* fractions were given once daily for 7 days. 1 h after the last treatment, rats was exposed to the FST. Immobility (**A**); climbing (**B**); and swimming (**C**) were recorded during the last 5 min in the FST. All data are mean ± SEM values (*n* = 8 per group). * *p* < 0.05, ** *p* < 0.01, and *** *p* < 0.001 significantly different from control group.

As shown in Figure 1, *M. alba* EtOAc fraction at the dose of 100 mg/kg induced a decrease in immobility time (*p* < 0.01) with a concomitant increase in both climbing (*p* < 0.05) and swimming (*p* < 0.05) times when compared with the control group. No significant differences were found in *M. alba* *n*-BuOH fractions treated groups.

### 2.2. Effects of M. alba Ethyl Acetate Soluble Fraction on Serum Corticosterone and Glucose Levels

It has been reported that FST exposure activates HPA axis and produces alterations in serum corticosterone and glucose levels [18,19].

**Figure 2:**
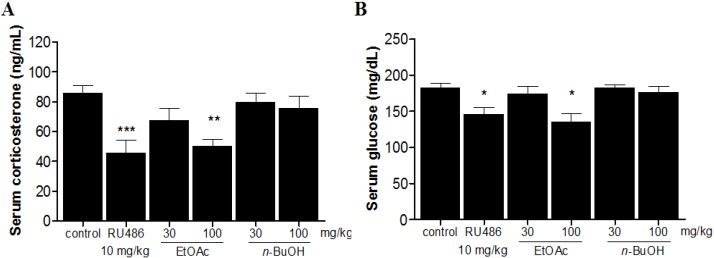
Effect of *M. alba* fractions on serum corticosterone (**A**) and glucose (**B**) levels. Data are mean ± SEM values (*n* = 8 per group). * *p* < 0.05, ** *p* < 0.01, and *** *p* < 0.001 significantly different from control group.

As shown in Figure 2, treatment with *M. alba* EtOAc fraction at the dose of 100 mg/kg significantly reduced the serum corticosterone (*p* < 0.01) and glucose (*p* < 0.05) levels compared with the control group, respectively.

### 2.3. Effects of M. alba Ethyl Acetate Soluble Fraction on c-fos Expression in Hippocampus and Hypothalamic PVN

To examine whether *M. alba* EtOAc fractions affect neural responses in the rats exposed to the FST, c-fos immunohistochemical density was measured in the hippocampus and hypothalamic PVN region. Since c-fos is an immediate early gene and rapidly induced by FST in relevant brain area such as hippocampus and hypothalamic PVN regions [20].

**Figure 3:**
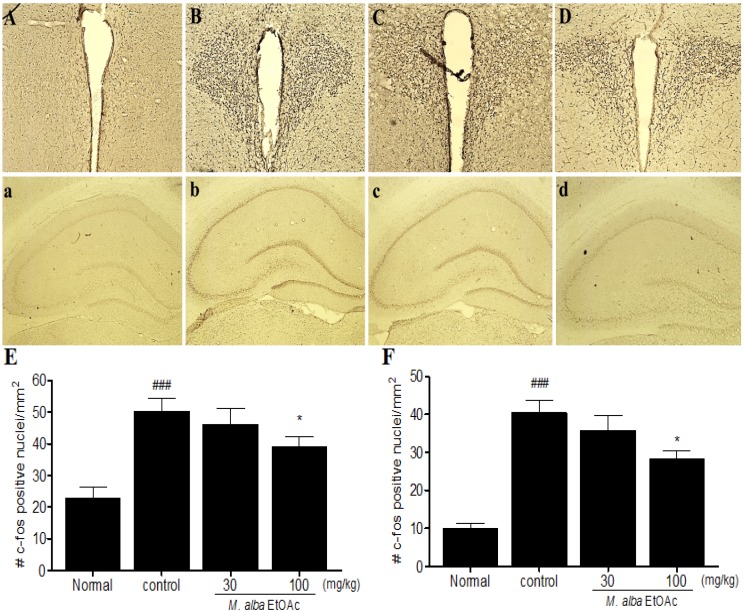
Effects of *M. alba* EtOAc fractions on c-fos expression in the hippocampus and PVN region. Representative photomicrographs are showing c-fos immunostained nuclei in the PVN of normal (**A**); control (**B**); *M. alba* EtOAc fraction 30 mg/kg (**C**); and *M. alba* EtOAc fraction 100 mg/kg (**D**); and in the hippocampus of normal (A); control (B); *M. alba* EtOAc fraction 30 mg/kg (C); and *M. alba* EtOAc fraction 100 mg/kg (D). Mean ± SEM values of c-fos immunostained nuclei in the hypothalamic PVN (**E**) and hippocampus (**F**). * *p* < 0.05, significantly different from the control group. ^###^ *p* < 0.001, significantly different from the normal group.

Animals exposed to FST and treated with vehicle displayed greater c-fos activation in the hypothalamic PVN and hippocampus (Figure 3B,b). A significant inhibition of c-fos immunoreactivity was observed in the group that was treated with *M. alba* EtOAc fraction at the dose of 100 mg/kg (Figure 3D,d).

### 2.4. Discussion

Our results demonstrated that 7 days of treatment with the ethyl acetate soluble fraction of *M. alba* significantly decreased the immobility time in rats exposed to the FST. Also the ethyl acetate soluble fraction of *M. alba* led to decreased HPA axis response to the stress, as indicated by an attenuated corticosterone response and decreased c-fos immunoreactivity in the hippocampus and PVN.

Animal models of depression play an important role in the scientific screening and evaluation of antidepressants. After an initial period of struggling, animals become immobile, resembling a state of despair and mental disorder. The state of immobility in the FST is reported to be similar to human depression and can be reversed with treatment of antidepressant drugs [21]. The present results showed that ethyl acetate soluble fraction of *M. alba* was effective in producing significant antidepressant-like effects in these model, especially at the dose of 100 mg/kg. RU486 (mifepristone), as a positive control, possesses potent antagonistic activity against glucocorticosteroid action [22]. These reports support the notion that GR antagonists may be useful as antidepressants for the treatment of depression, via the regulation of the HPA axis [23]. Wulsin *et al.* reported that treatment with RU486 decreased immobility behavior in the FST, consistent with anti-depressant action via alterations in key limbic circuits mediating central nervous system (CNS) stress responses [24]. Consistent with our findings in the RU486 treated group, ethyl acetate soluble fraction of *M. alba* might have antidepressant-like activity as tested in the FST.

The antidepressant-like activity of the ethyl acetate soluble fraction of *M. alba* was confirmed via the quantitative analyses of c-fos immunoreactivity and of the activity of the HPA axis, which are associated with high corticosterone production. Previous studies involving acute or chronic stress states have demonstrated that profound alterations in GR mRNA expression are closely associated with elevated corticosterone production and c-fos expression [25]. Antidepressant drug, including selective serotonin reuptake inhibitors (SSRIs), compensate impaired feedback inhibition by regulating GR levels in hippocampal regions [26]. Consistent with this idea, we found that treatment with ethyl acetate soluble fraction of *M. alba* at the dose of 100 mg/kg alleviated not only the increase in c-fos-positive cells in the hippocampus and hypothalamic PVN associated with stress-induced depression, but also the HPA axis response to stress. Our findings suggest that the ethyl acetate soluble fraction of *M. alba* reverses the down-regulation of hippocampal GR expression in rats exposed to stress.

## 3. Experimental

### 3.1. Preparation of M. alba Fractions

Dried root bark of *M. alba* (10 kg, Kapdang, Seoul, Korea) was prepared after immersion in 80% methanol (170 L) and shaking at room temperature for 24 h. The process was repeated, and the extracts were combined and filtered through a membrane filter (0.45 µm; Millipore, Billerica, MA, USA). After removing the solvents via rotary evaporation, the remaining extracts were vacuum dried to a yield of about 17% (w/w). The extracts were then separated successively with ethyl acetate (EtOAc) (580 g), *n*-BuOH (114 g) and H_2_O (1 kg).

### 3.2. Animals and Treatments

8-week-old male Wistar rats (Japan SLC, Inc., Hamamatsu, Japan) weighing 180–210 g were housed at two rats per cage under a controlled temperature (23 ± 1 °C) with a 12 h light/dark cycle (lights on at 07h00 and lights off at 19h00). The rats were allowed at least 1 week to adapt to their environment before the experiments. All animal experiments were carried out according to the guidelines of the Korea Food Research Institutional Animal Care and Use Committee. The rats were randomly divided into six groups (n = 8 per group); normal, control, RU486 (10 mg/kg), *M. alba* ethyl acetate fractions (30 and 100 mg/kg) and *M. alba* *n*-BuOH fractions (30 and 100 mg/kg) were orally administered every day for 7 consecutive days. *M. alba* fractions were dissolved in distilled water. The vehicle groups; normal (non-stress) and control (stress) were administered the vehicle solution (distilled water, 1 mL/kg, p.o.) using the same schedule of administration. RU486 (Sigma, St. Louis, MO, USA) dissolved in 50% sesame oil was used as a positive control of antidepressant activity [27]. On day 7, 1 h after the final administration, the rats were exposed to the behavioral experiments.

### 3.3. Forced Swimming Test (FST)

The FST was carried out as previously described [28]. Briefly, rats were placed individually in a transparent Plexiglas cylinder (50 cm × 20 cm) filled to a 30 cm depth with 23–25 °C water. Rats were forced to swim for 15 min, and the immobility time during the last 5 min was measured by a blinded observer. Rats were considered immobile when they ceased struggling, remained floating motionless, and only made those movements necessary to keep their heads above the water [29].

### 3.4. Corticosterone Assay and Biochemical Analysis

Blood samples were collected via the abdominal aorta after the FST. The serum samples were prepared by centrifugation of the collected blood samples (1013 g for 15 min), then stored at −80 °C for biochemical determinations. The serum levels of corticosterone were measured using commercially available EIA kits (R&D Systems, Inc., Minneapolis, MN, USA).

### 3.5. Immunohistochemistry

Rats were sacrificed following the FST test, and their brains were fixed through the ascending aorta with 0.9% saline, followed by 500 mL of cold 0.1 M phosphate buffer (PB) containing 4% paraformaldehyde (PFA). The fixed brains were cut into 30 µm sections on a cryostat (CM1850; Leica, Heidelberg, Germany). Immunohistochemistry staining was performed on 30 µm sections using polyclonal antibodies specific for c-fos (1:500 dilution; Santa Cruz Biotechnology, Santa Cruz, CA, USA), followed by exposure to an biotinylated anti-rabbit antibody (1:500 dilution; Vector Labs, BA1000, Burlington, ON, Canada). The sections were reacted with an avidin–biotin–peroxidase complex kit (Elite ABC kit; 1:50 dilution, Vector Laboratories) at room temperature for 60 min. The avidin–biotin complex was visualized with 0.05% 3, 3-diaminobenzidine (DAB; Sigma) and 0.02% H_2_O_2_.

### 3.6. Statistical Analysis

Data analyses were performed using one-way analysis of variance (ANOVA), followed by Tukey’s post hoc test, using Prism 5 (GraphPad Software, Inc., San Diego, CA, USA) for multigroup comparisons. All data are presented as the mean ± standard error (SEM). Significance was set at *p* < 0.05.

## 4. Conclusions

In conclusion, our results demonstrated that treatment with the ethyl acetate soluble fraction of *M. alba* significantly decreased the immobility time in rats exposed to the FST, and decreased the HPA axis response to the stress, as indicated by an attenuated corticosterone response and decreased c-fos immunoreactivity in the hippocampal hypothalamic PVN region.
